# JigCell Run Manager (JC-RM): a tool for managing large sets of biochemical model parametrizations

**DOI:** 10.1186/s12918-015-0237-0

**Published:** 2015-12-24

**Authors:** Alida Palmisano, Stefan Hoops, Layne T. Watson, Thomas C. Jones, John J. Tyson, Clifford A. Shaffer

**Affiliations:** Department of Computer Science, Virginia Tech, 2202 Kraft Drive, Blacksburg, 24060 VA USA; Department of Biological Sciences, Virginia Tech, 1405 Perry Street, Blacksburg, 24061 VA USA; Virginia Bioinformatics Institute, 1015 Life Science Circle, Blacksburg, 24061 VA USA; Department of Mathematics, Virginia Tech, 225 Stanger Street, Blacksburg, 24061 VA USA

**Keywords:** Systems biology, Biological networks, Mathematical modeling, Chemical reaction systems, COPASI, SBML, Software, Model editor, Parameter hierarchy, Mutants

## Abstract

**Background:**

Most biomolecular reaction modeling tools allow users to build models with a single list of parameter values. However, a common scenario involves different parameterizations of the model to account for the results of related experiments, for example, to define the phenotypes for a variety of mutations (gene knockout, over expression, etc.) of a specific biochemical network. This scenario is not well supported by existing model editors, forcing the user to manually generate, store, and maintain many variations of the same model.

**Results:**

We developed an extension to our modeling editor called the JigCell Run Manager (JC-RM). JC-RM allows the modeler to define a hierarchy of parameter values, simulations, and plot settings, and to save them together with the initial model. JC-RM supports generation of simulation plots, as well as export to COPASI and SBML (L3V1) for further analysis.

**Conclusions:**

Developing a model with its initial list of parameter values is just the first step in modeling a biological system. Models are often parameterized in many different ways to account for mutations of the organism and/or for sets of related experiments performed on the organism. JC-RM offers two critical features: it supports the everyday management of a large model, complete with its parameterizations, and it facilitates sharing this information before and after publication. JC-RM allows the modeler to define a hierarchy of parameter values, simulation, and plot settings, and to maintain a relationship between this hierarchy and the initial model. JC-RM is implemented in Java and uses the COPASI API. JC-RM runs on all major operating systems, with minimal system requirements. Installers, source code, user manual, and examples can be found at the COPASI website (http://www.copasi.org/Projects).

## Background

Developing a model with its initial list of parameter values to reproduce a specific behavior is just the first step in the process of modeling biochemical systems. Often a model must be parameterized in many different ways to account for a set of related experiments carried out on an organism, and the results simulated and plotted for every situation. For example, a budding yeast cell cycle model [1] accounts for more than 120 different mutants, which are listed as changes of specific parameter values on the model website [http://mpf.biol.vt.edu/research/budding_yeast_model/pp/]. The published model is available on Biomodels [2] as a single SBML file, parameterized for simulating wild-type cells [https://www.ebi.ac.uk/biomodels-main/BIOMD0000000056]. Each mutation can be simulated by manually changing specific parameters and saving the model to a new SBML file. This approach creates a large collection of files that is hard to maintain, use, and almost impossible to share. Any scientist interested in reproducing the results of the original paper must dedicate a significant amount of time to recreate each variant of the model, starting from the initial model file. This process is both time-consuming and error-prone, because each file is an independent and separate entity. Discovering a mistake in any file may compromise the entire batch of files, since it is very likely that different files were created by a copy-paste approach that spreads the error.

An important observation is that for many modeling scenarios, parameter changes between different mutants are minimal and often connected to each other. A single gene knockout mutant, for example, may just set the values of a few kinetic rate constants to zero. A double mutant, where both gene A and B have been knocked out, might contain the union of changes made in the single gene knockout mutant A and single gene knockout mutant B. While describing these mutants using natural language is straightforward, encoding them in formal languages (like SBML) is hard, due to the lack of tools that deal with more than one model/parameterization at the same time.

The need for better tools and approaches to tackle higher-order modeling issues in systems biology and systems medicine has been recognized by Wolkenhauer, et al. [3], who identified a need to develop “workflows for modeling, including computational tools that support data management, model construction and analysis” and “dedicated modeling workflows for the integration of data and models”. To address this need to develop a model in synergy with its parameterizations, we created a new version of the JigCell Run Manager tool [4] that allows modelers to organize, maintain, simulate, plot, and export hierarchical parameterizations of biochemical models.

### Related work

The importance of linking a model with its parameterizations is acknowledged by the SBML community through the ongoing effort of the Simulation Experiment Description Markup Language (SED-ML) [5]. Instructions for simulation, analysis, and visualization can be encoded in SED-ML and stored together with the model definition. However SED-ML does not provide means to hierarchically encode modifications nor it provides ways to detect/resolve conflicts that may arise when a parameterization inherits its values from multiple sources (see Section ‘Detecting and resolving conflicts’).

Currently there are many tools that can create, edit, and simulate SBML biochemical reaction models (e.g., COPASI [6], CellDesigner [7], Virtual Cell [8]). None of these collect hierarchical groups of parameterizations for a single model.

COPASI defines a “parameter sets” option to collect different parameter values together with the model. Unfortunately, each configuration is independent of the others, and parameterizations are not related to simulation or plotting configurations. These limitations make it cumbersome to work with parameterizations, since some mutants have special simulation or plotting requirements that need to be changed manually each time that particular mutant is to be simulated. Moreover, the lack of hierarchical relations among parameter sets makes it hard to see the relationships among simulations, such as simulations of single, double and triple mutants.

Snoopy [9], a Petri net editing and simulation tool, has limited support for parameter sets, similar to COPASI. Snoopy can store multiple configurations for a model, but parameter value inheritance between configurations is not supported. This approach offers little help in situations where the number of simulations is large (e.g., hundreds of mutants), because each configuration has to be manipulated independently.

The present work is based on a previous version of a run-manager tool [4]. Major additions and changes have been implemented, including a flexible way to inherit parameter values, conflict detection, the use of the COPASI API to seamlessly provide import/export capabilities, and integration with COPASI simulation tools. Additionally, JC-RM’s hierarchical visualization of experiments simplifies the management of collections of model parameterizations by enabling the modeler to visualize and simulate only the relevant parts of configuration ensembles.

## Results and discussion

JC-RM is an extension to our modeling editor (MSMB) [10] that allows the modeler to define hierarchies for parameter values, simulations, and plot settings, and to save them together with the initial model.

Key features of this tool include the following.

- **Visual representation of configuration hierarchies**: JC-RM guides the user in creating a hierarchy of parameter configurations for a model. These configurations are displayed as a directed acyclic graph (DAG). Use of a DAG is motivated by the notion of inheriting a small number of changes from an existing configuration. For each node, the values of chosen parameters can be changed to new numerical values or to algebraic expressions involving values from ancestors. This makes it easy to define and maintain big collections of parameter configurations. New values can be assigned to global quantities, initial amounts of species and compartment sizes. JC-RM supports separate DAGs for a model’s parameter values, for the associated simulation settings, and for multiple plot settings for various output requirements.
- **Algebraic expressions involving ancestors’ values**: Any node in the hierarchy can refer to values of ancestor nodes to determine its local values. For example, the user can assign the parameter k1 in one node as two times the same parameter in the parent plus four times the same parameter in the grandparent (k1 = 2*k1@parent + 4*k2@grandparent). In this way if a change is made to the value of k1 in the parent or grandparent, this would be carried out seamlessly through the descendant nodes, simplifying the management of large, complex hierarchical structures.
- **Conflict detection**: Defining a configuration as a child of multiple parent nodes might result in conflicts if parents redefine a parameter to different values. JC-RM detects and communicates these conflicts to the modeler, who can resolve them either by assigning a new local value to the parameter or by choosing the value from one specific parent. Graphs with conflicts can be stored in the JC-RM internal format; however, the conflicts must be resolved before the graphs can be used for simulation or exported.
- **Preset graph layouts and other visual customizations:**JC-RM offers many automatic graph layout options. The look of the graph can be manually customized by moving the nodes on the screen and by assigning different colors to nodes and edges, with the resulting layout stored. Multiple views can be saved internally and/or exported to JPEG for publication purposes.
- **Running simulations internally or export to COPASI**: Once the settings for model parameter values, simulation conditions, and plotting instructions are consistent (i.e., without conflicting definitions), JC-RM allows the user to run simulations and see the results (e.g., time series or phase planes). Alternatively, the hierarchy of parameter values coupled with the basal simulation and plotting settings can be exported to COPASI and SBML (L3V1) for further analysis.

### JC-RM primary features

In this section we outline how JC-RM can be used in the everyday task of managing a model of a complex biochemical system. As a working example, we use a published model of the budding yeast cell cycle [1], available in SBML format in the Biomodel database (model 56, [2]). The SBML version in the Biomodel database is parameterized for simulation of the cell division cycle in wild-type budding yeast cells. However, the published model accounts for more than 100 different mutant strains of budding yeast. The collection of the parameter variations needed to model these different strains is listed at http://mpf.biol.vt.edu/research/budding_yeast_model/pp/.

**Fig. 1:**
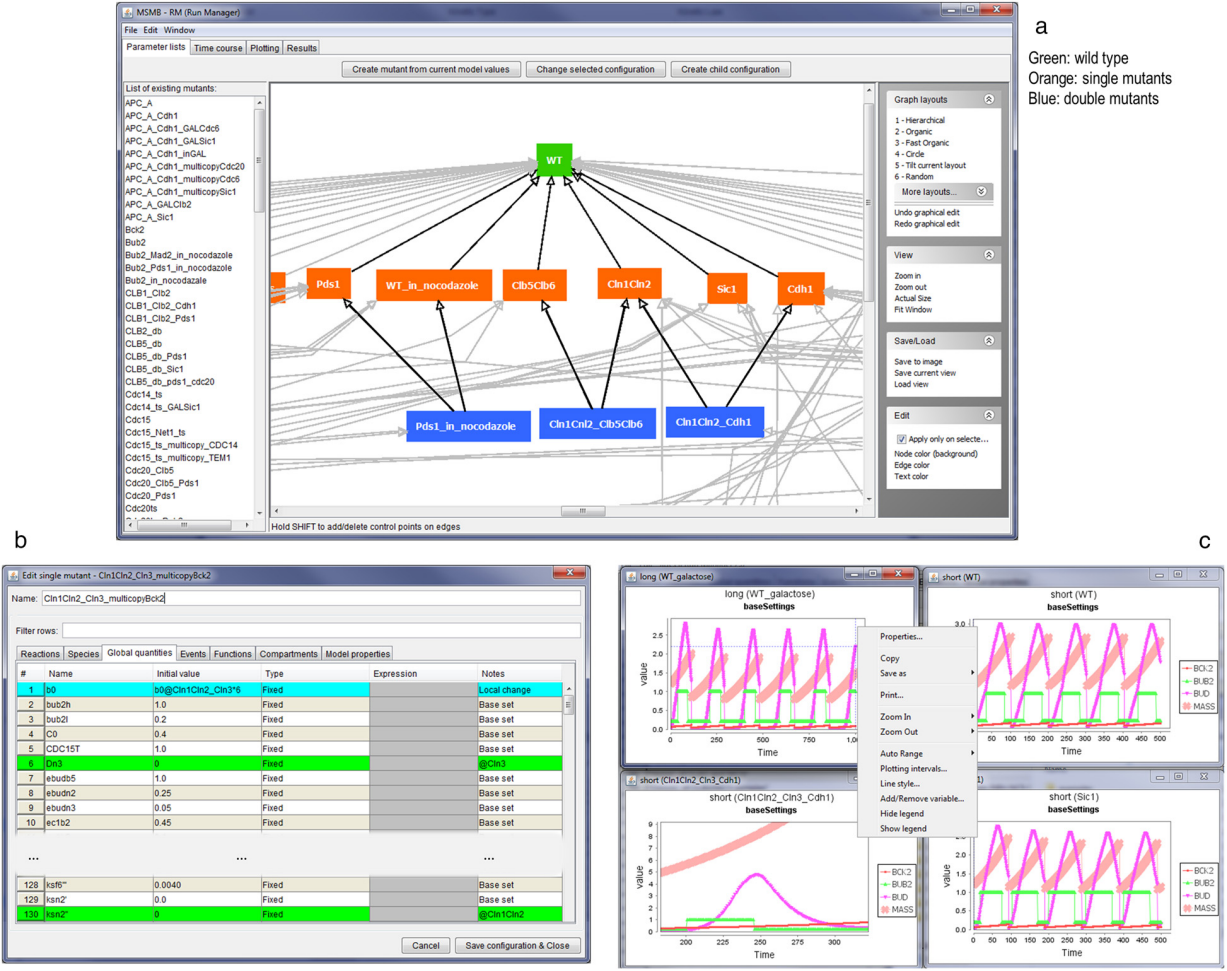
**a** The collection of parameter values is displayed in a DAG and in a list in the left portion of the main window. Edges in the graph point from children to parents. Green, orange, blue nodes are wild type, single mutant, double mutant configurations, respectively. **b** The content for one parameter value configuration. The origin of the value is presented in the *Notes* column (i.e., some values belong to the *Base set*, some are inherited from ancestor nodes denoted with @*ancestorName*, while others are local redefinitions). **c** Time series plots generated and presented by JC-RM. As shown in the context menu, many options are available to customize the look of a plot

The code for this example is available for download from the JC-RM website. For our example, we show how to start from the initial SBML model and add mutant parameterizations, along with simulation and plotting configurations. Repeating the same steps for each mutant will generate the final file available for download in the software package (depicted in Fig. 1). This file contains the 131 mutant configurations of the budding yeast cell cycle model by Chen, et al. [1].

### The parameters graph

The following steps are used to create the Parameter Lists graph.

1. The SBML model file (from https://www.ebi.ac.uk/biomodels-main/BIOMD0000000056) is loaded into the JC-MSMB editor, where the Reactions, Species, etc. that compose the original model are displayed. This is the base model used to build the hierarchy of parameterizations.
2. Opening JC-RM displays the main window. It contains independent but connected graphs, each representing a different aspect of the model parameterization (Model values, Time course, Plotting,...). The first time the window is opened, each tab will be empty. Once graphs have been added to the model, the user can save the model with the parameterization through the “Save to.msmb" option. At any future time, the user can open the saved.msmb file and continue working on either the model or its parameterization.
3. To create the first node of the DAG, from the “Parameter lists” tab the user clicks the “Create mutant from current model values” button. JC-RM will ask to give this configuration a name. Since the current model holds the parameters for the wild type in glucose, in our example we call the node WT_glucose. The newly created node will appear both in the graph view and in the alphabetically ordered list of defined configurations (in the left portion of the window). As imported from Biomodels, the model already contains the right numerical values for modeling the wild type in glucose, we don’t need to customize the content for this node. So we can move to defining new nodes in the Parameter list graph.
4. A new mutant can be defined as a child of any node in the graph. This allows the child configuration to inherit all the values of the parent (or parents), possibly redefining some specific values in the local configuration. More complete examples where multiple parents are used for the mutant definition (and any resulting conflicts are detected) are presented in section ‘Detecting and resolving conflicts’. To create a child configuration, select the root node in the graph (WT_glucose) and then click the “Create child configuration” button. The user will be asked to give this new configuration a name (the name must be unique within the graph). We will call the new node WT_galactose. The newly created node will appear both in the graph view (as a node connected to its parent) and in the list of all defined lists.
5. To make some parameter values for WT_galactose different from WT_glucose, the new configuration needs to be changed (Fig. 2). To do so, the user can edit the node s/he wants to change by either double clicking on the node or by clicking the “Edit mutant configuration” button. This will open a pop-up window in which the various tables defining the models are shown. The WT_galactose configuration differs from the WT_glucose in the value of the “mdt" global quantity (150 instead of 80). The user can find the mdt element in the proper tab, double click on the “Initial Value” cell of mdt and assign the new value. Once the change has been made, the fact that the value coming from the parent has been overwritten by the new value is visually identified by the color of mdt’s row, as well as through a new label shown in the “Notes” column (i.e., “Local change"). This label helps the user to keep track of the changes. The “Notes” column can be used as a filter with the search/filter box.

**Fig. 2:**
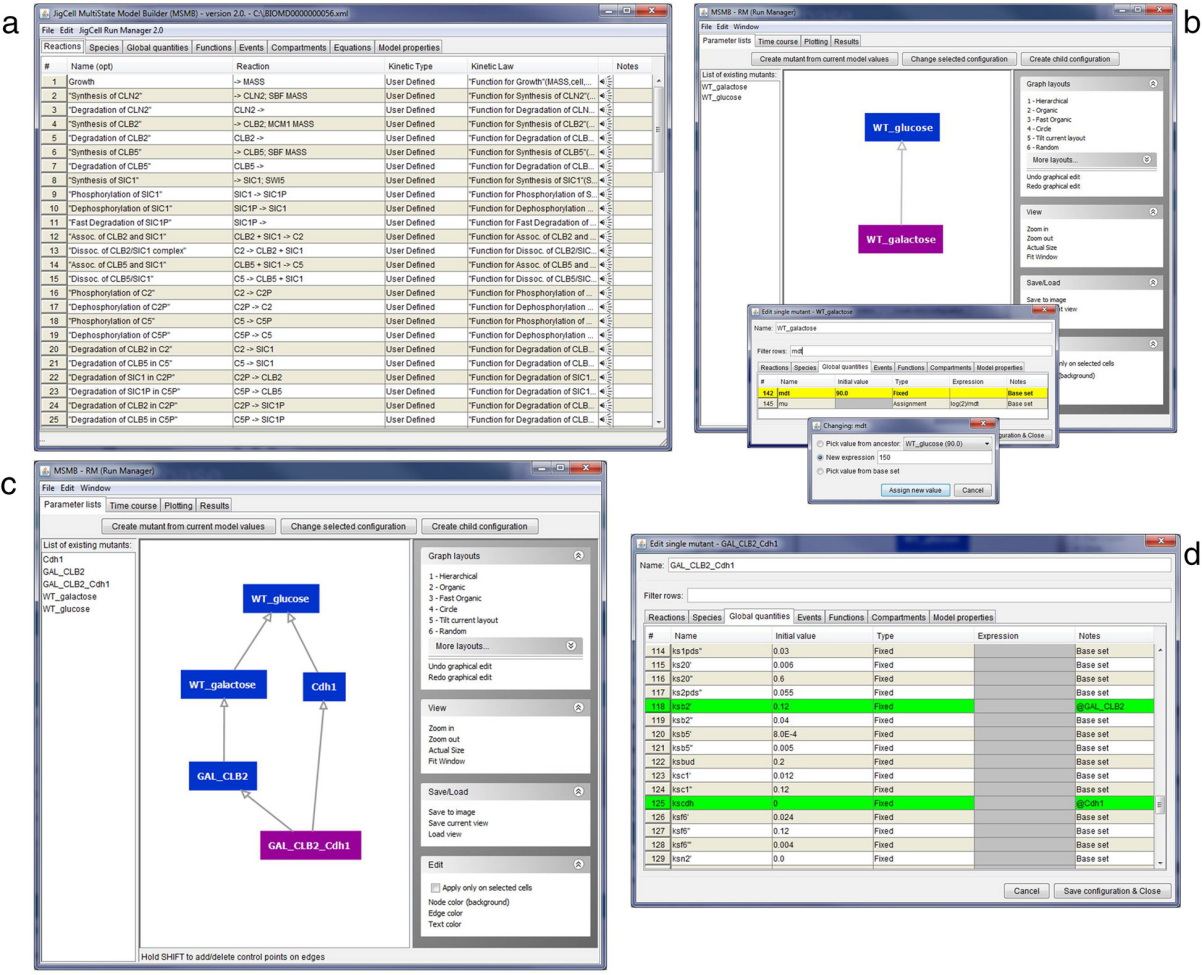
Steps to create a parameter list graph from an initial model file. **a** The SBML model is loaded in the editor and all its component are displayed in the tables. **b** The JC-RM interface contains two nodes representing mutant configurations of the initial model setup. For details on how to create those nodes, refer to the text. To edit specific parameter values, the modeler can use the filtering capabilities of the editor. A new value can be assigned in 3 different ways: more details on this can be found in the text. **c** A more complex DAG connects different parameter configurations. **d** Of particular interest, the node called ‘GAL_CLB2_Cdh1’: it inherits from two parents and the specific values inherited from each parent are clearly marked in the tabular view
6. Repeating these steps, the user can add all mutant configurations. To define a “double mutant" configuration, the user selects multiple nodes from which the child configuration will inherit, and then clicks the “Create new configuration" button. This new mutant will contain the union of the changes of all the parents. (See ‘Detecting and resolving conflicts’ for a discussion on resolving any conflicts that might result).

In all graphs, newly created nodes are automatically positioned on the screen. Users can reposition nodes manually with the mouse, or they can use one of the alternative automatic graph layout options provided. Node and edge colors can be changed to suit the needs of the modeler. Colors may be useful to visually group mutants of similar nature, or to quickly identify configurations of interest that are incomplete or need attention.

Any node in the hierarchy can be deleted and a warning is displayed to the user before the action is completed. The warning displays the names of all the nodes that may be affected by this deletion (i.e., the descendants that inherit values from the deleted node). The recommended practice is to take note of this list and make sure that no undesired effect is propagated. There are different possible scenarios:

1. The child inherits the parameter values of ancestor nodes without local changes. If one of the ancestors is removed, the parameter values of the child will be determined by either other ancestors node still connected, or by the “base set”, which is the parameter value of the initial model from the JC-MSMB interface.
2. The child overwrites the value of a parameter, making explicit reference to the deleted parent. In this case a “inconsistency” warning is displayed for the node, and the user won’t be allowed to run simulations of this set until the inconsistency is resolved.

Deletion of single parameters within a node configuration is not currently supported in JC-RM because this represents a structural change in the model which may have hard to handle consequences in terms of model management (e.g., leaving dangling references in rate laws, assignments, etc.). The effect of deleting a parameter, however, can be easily achieved by setting its value to zero. This corresponds to knocking out a specific pathway or reaction, without disrupting the structure of the entire hierarchy of parameter configurations.

### The time course settings graph

The idea of defining changes in a hierarchical way can be applied not just to model parameters but also to simulation settings. A given mutant might need to be simulated with runtime parameters different from the others. For example, the budding yeast cell cycle model has mutants that represent the organism when grown in different media. This is modeled by slowing the cell cycle machinery. Hence in a fixed time period, fewer complete cycles can be observed. The modeler might therefore wish to set a different total time period in the simulation settings for these slow mutants, so that the overall number of cycles is comparable to the fast mutants, without slowing down the entire process by increasing the simulation time for all of the mutants. In principle, this process is similar to describing changes in model parameters, because again only one element in the simulation settings (the end time in this case) needs to change.

JC-RM allows the user to define a DAG for simulation settings under the “Time course” tab. The layout of the tab and the process for building the graph is similar to the “Parameter lists” tab. When editing a simulation graph node, the user sees a window that shows relevant information that a simulation may need to customize. Each defined simulation configuration can be applied to specific mutants defined in the Parameter Lists tab.

**Fig. 3:**
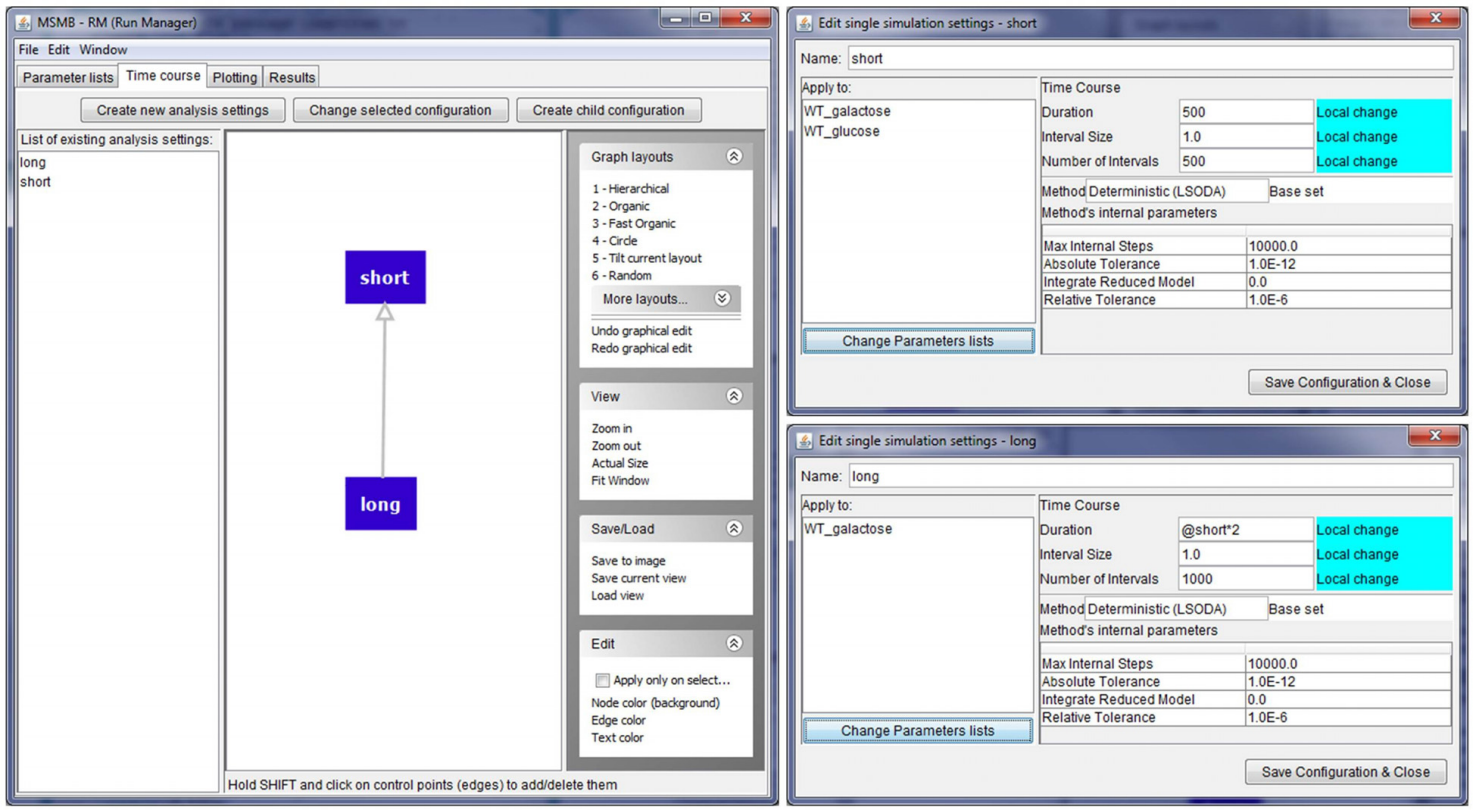
Initializing time course settings. (*left*) A graph containing two Time course nodes (called short and long). The node called ‘long’ inherits the settings from its parent, redefining some quantities with new values (local changes). (*right top*) ‘Short’ simulation configuration details. Duration and interval size are changed with respect to the basic default configuration determined by JC-RM. Other values, like the kind of simulation engine with its internal parameters, are left unchanged. (*right bottom*) ‘Long’ simulation configuration details. Duration and interval size are changed with respect to the inherited values. Since the two nodes are connected, the descendant values can be expressed in terms of any ascendant parameter, making the expression of the duration in this node highly flexible. Each Time Course node applies to a specific list of ‘Parameter sets’ as shown in the left portion of each window: in this case the short configuration is applied to both WT and WT_galactose while the long configuration is applied only to the WT_galactose node

In Fig. 3, two simulation settings are shown. One is called “short” (with duration parameter of 500) and another “long” (with duration parameter 1000). In the example, the long configuration is applied only to the WT_galactose node, while the short one is applied to both.

### The plot settings graph

The user might wish to present specific mutants in plots with different criteria. For example, in a mutant where the gene for the Cdh1 protein has been knocked out, the time course of the protein Cdh1 could be omitted (since it should be constantly zero). Of course, the collection of mutants that need a specific configuration for plot settings is not necessarily related to the hierarchy defined for parameter settings or simulation settings. So JC-RM provides the “Plotting” tab for specifying the relationships between various plotting configurations. This tab follows the same process for defining relationships as is used for model parameters and simulation settings. Double clicking on a node in the plot settings graph opens a pop-up window where the user can select specific values for the current plotting settings. The user will choose any of the model variables (or time) as the X axis, and one or more of the other variables for the Y axis. It is also possible to customize the appearance for each line in the plot (i.e., color, thickness, symbol and plotting interval), and customize other general layout options for the XY plot (e.g., font and color of the title, x/y labels, plot background color, etc.).

While JC-RM offers the opportunity to define hierarchies for simulation and plot settings, it is not mandatory to do so. The default is to have a single group of settings for all mutants.

### Detecting and resolving conflicts

The hierarchical representation for values in graph nodes allows for setting a mutant’s parameter values to numerical values or to algebraic expressions involving values from ancestor nodes. Defining a configuration as a child of multiple parent nodes may result in conflicts, if multiple parents redefine a given parameter to different values. JC-RM detects and communicates these conflicts to the modeler, who is expected to resolve the conflict either by assigning a new local value to the parameter, or by choosing the value from one specific parent. Graphs with conflicts cannot be used to initiate simulations, nor can they be exported to COPASI or SBML. They may, however, be stored in JC-RM’s internal format so that the user does not lose work.

A scenario with conflicts is depicted in Fig. 4, where two separate configurations are defined (WT1 and WT2). Each has a local assignment for parameter k1, with values 2 and 4, respectively. When creating a third configuration named “M” as the child of WT1 and WT2, JC-RM detects a conflict in the value of k1. This issue is presented to the user through the use of dashed edges. Double clicking on the node displaying the conflict (M) will show to the user the specific parameter for which the conflict exists. At this point, the user must decide how to resolve the conflict. Three options are available.

1. Pick a value from a specific parent, selected using a drop down menu.

**Fig. 4:**
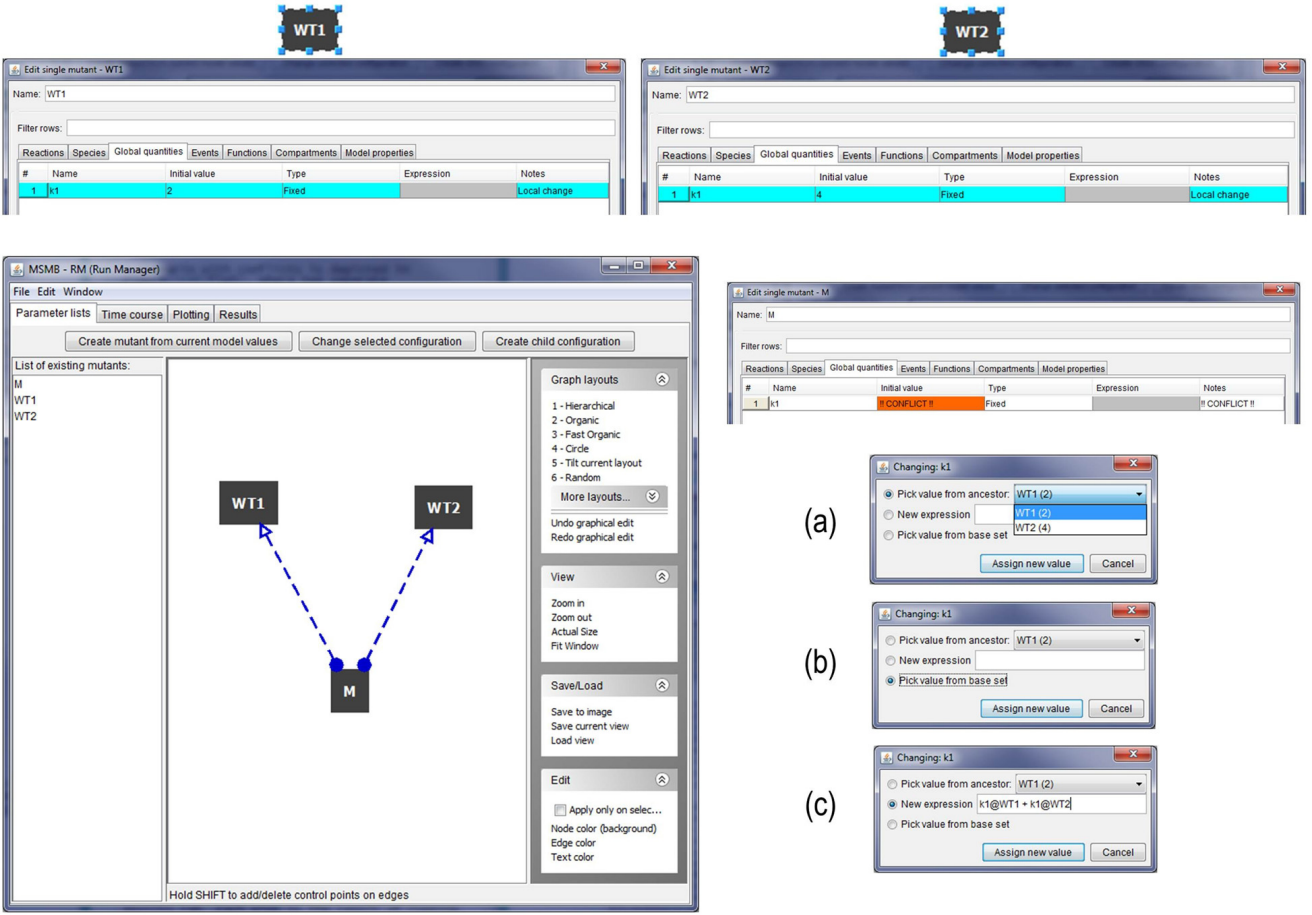
Conflict detection and resolution. (*top*) two nodes are created to generate a conflict. The two nodes locally assign a different value to the same parameter k1. (bottom left) Once the third node M is created as child of both WT1 and WT2 JC-RM detects the conflict in assigning a value to k1 in M and displays the problem as dashed connecting arrows in the graph. (*bottom right*) Checking the content of the M node, the conflict in the specific value of k1 is clearly marked. Three options are presented for resolving the conflict: **a** pick a value from a specific parent, **b** pick the value from the base set or **c** assign a new value from a typed expression. The algebraic expression can contain references to ancestor nodes
2. Pick the value from the original base set.
3. Assign a new value, which can be either a number or an algebraic expression that involves values from the parents. Such an expression will use the syntax “parameter_name@parent_name”. In the example from Fig. 4(c), M the sum of the value for k1 in WT1 and k1 in WT2.

Once the conflict has been resolved, the corresponding graph will display the edge between the node and its parents as a regular line.

### Running a simulation and visualizing the results

Once the settings for model parameter values, simulation conditions, and plotting options are consistent (i.e., without conflicting definitions), JC-RM allows the user to run simulations and see the results (e.g., time series or phase planes). When simulations are run within JC-RM, the user can choose which mutant(s) to simulate. Alternatively, the model can be exported to COPASI and SBML (L3V1) for further analysis. Note that while SBML Level 3 supports multiple parameter sets (so all of the various mutants can be represented), SBML only supports a single specification for simulation and plot settings. The user can select which to export.

**Fig. 5:**
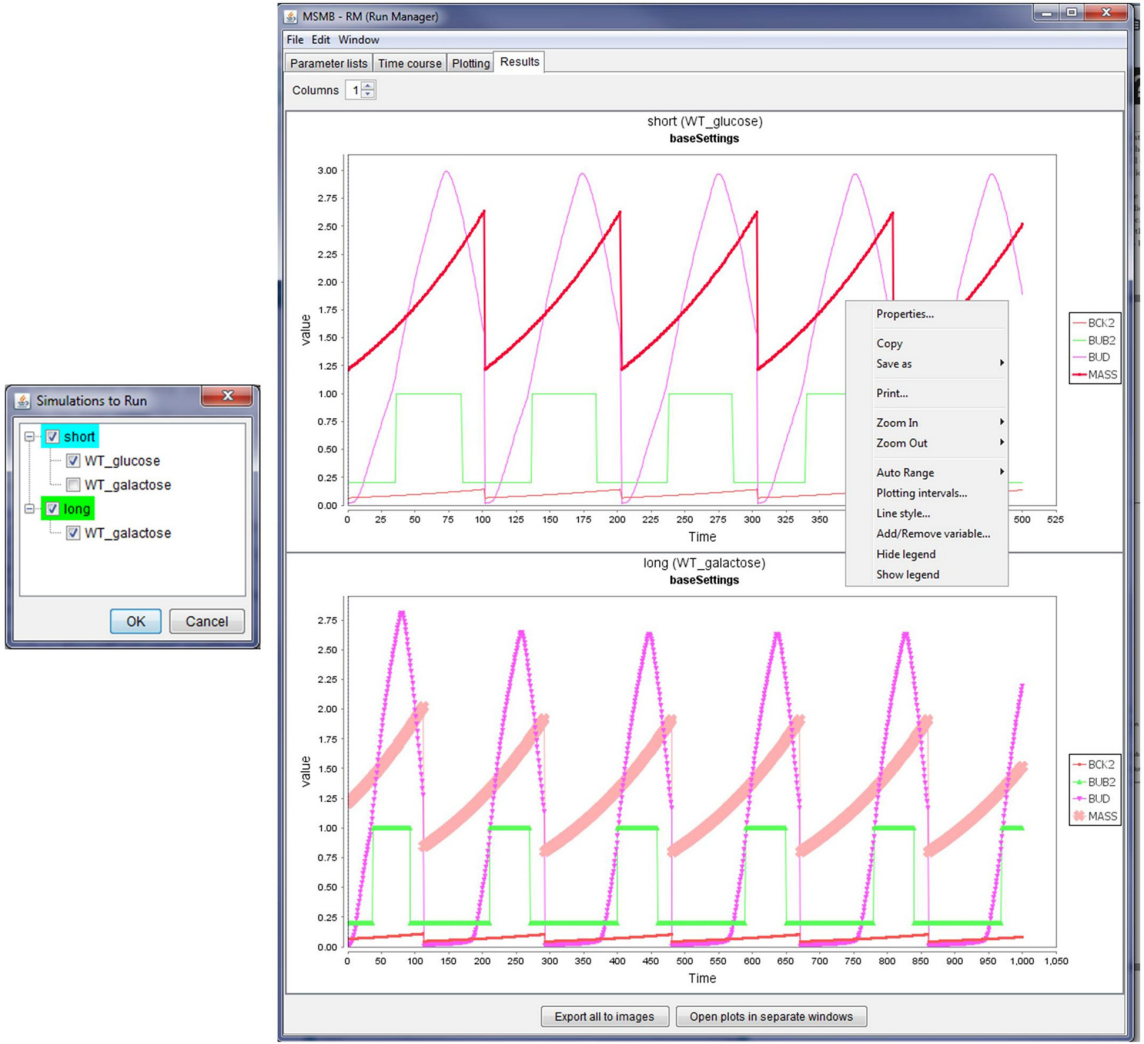
Running a simulation and visualizing the results. (*left*) When the user decides to run the simulation within JC-RM, a popup provides the modeler the choice to run specific nodes (shown as alphabetically sorted root nodes in the tree structure) or specific mutant configurations within each configuration. This option is useful when some mutants require longer simulation time and the modeler wants to check the behaviour of specific mutants. Colors identify nodes where all the mutant configurations are selected (green), some are omitted (cyan) or none is selected (white background, not shown). (*right*) Time course plots are displayed in the Results tab. Each plot is the result of running a selected Time Course node, with a specific parameter set configuration and the X/Y plot created according to the specification in the Plot graph. Each plot can be further customized, printed and saved using a contextual menu

If the simulations are run within JC-RM, the results can be viewed, as in Fig. 5. Each plot can be separately customized, using options accessible from the right-click menu (zoom, change line colors, save it as an image, add/remove variables, etc.). The plots are embedded in the main window, but for easy comparison, the option “Open plots to separate windows” is available. This causes each plot to be displayed in a separate window that can be repositioned or maximized on the user screen. Closing each plot window will cause that plot to go back to its embedded state. The “Window" menu helps the user to bring to the front a selected plot.

## Conclusions

Developing a model with its initial list of parameter values is just the first step in modeling a biological system. Models are often parameterized in many different ways to account for mutations of the organism or sets of related experiments performed on the organism. During the life cycle of a modeling project, the model may need frequent changes, values need to be updated, specific mutants need adjustment, etc. If these operations must be performed in a context where each parameterization is a separate and independent file, the modeling process becomes difficult, cumbersome and error-prone. Since modeling efforts are typically carried out by a team of researchers, coordinating the exchange and updates of hundreds of model files among team members is even more chaotic. If the research team follows the traditional practice of sharing only one model file when the work is finalized and published, then any scientist interested in the model must start almost from scratch and expend considerable effort to reproduce the simulations in the publication.

By allowing modelers to define a hierarchy of parameter values, simulation settings, and plot settings, and to couple these hierarchies with the initial model, JC-RM enables computational modeling in two critical ways:

1. By supporting everyday management of a large model, complete with its parameterizations.
2. By facilitating the sharing of this information before and after publication.

Future developments for JC-RM include the addition of analysis tools for data other than time course simulations (e.g., parameter estimation tasks) that require different experimental data to be associated with different lists of parameter values. Another valuable future addition would be the implementation of direct export capabilities to SED-ML [5]. SED-ML is a language developed by the SBML community to standardize the coupling of model parameterization and simulation settings. Its purpose is to support exchange of models with reproducible results. The current SED-ML specification includes elements like “List of Models”, “Changes” and “Simulations” that quite nicely map to concepts implemented in JC-RM. While the concepts are similar, they are not used in the same way in our software, so investigating the direct import/export capabilities is beyond the scope of this work and it has been deferred for a follow-up version of JC-RM. As active members of the SBML community, we hope to contribute to further developments of this standard.

## Availability and requirements

1. Project name: JC-RM
2. Project home page: http://www.copasi.org/Projects
3. Operating system(s): Platform independent
4. Programming language: Java
5. Other requirements: Java 6.0 or higher
6. License: Artistic License 2.0

## Implementation

JC-RM is implemented in Java and uses the COPASI API [6]. JC-RM runs on all major operating systems, with minimal system requirements. Installer, source code, user manual, and examples can be found at the COPASI website (http://copasi.org/Projects/).
